# Pulsed Field Ablation of Atrial Fibrillation: A Novel Technology for Safer and Faster Ablation

**DOI:** 10.3390/biomedicines12102232

**Published:** 2024-09-30

**Authors:** Alejandro Carta-Bergaz, Gonzalo R. Ríos-Muñoz, Pablo Ávila, Felipe Atienza, Esteban González-Torrecilla, Ángel Arenal

**Affiliations:** 1 Department of Cardiology, Gregorio Marañón Health Research Institute (IiSGM), Calle Dr. Esquerdo 47, 28007 Madrid, Spain; gonzalo.rios.munoz@secardiologia.es (G.R.R.-M.); pabloavila@salud.madrid.org (P.Á.); felipe.atienza@salud.madrid.org (F.A.); etorrecilla@telefonica.net (E.G.-T.); arenal@secardiologia.es (Á.A.); 2 Centre for Biomedical Research in Cardiovascular Disease Network (CIBERCV), Instituto de Salud Carlos III, Calle Monforte de Lemos 3-5, 28029 Madrid, Spain; 3 Department of Bioengineering, Universidad Carlos III de Madrid, Escuela Politécnica Superior, Avda de la Universidad 30, 28911 Madrid, Spain; 4 Medicine School, Universidad Complutense de Madrid, Plaza de Ramón y Cajal s/n, 28040 Madrid, Spain

**Keywords:** atrial fibrillation, catheter ablation, electroporation, pulsed field ablation

## Abstract

Atrial fibrillation (AF), the most common arrhythmia, is associated with increased morbidity, mortality, and healthcare costs. Evidence indicates that rhythm control offers superior cardiovascular outcomes compared to rate control, especially when initiated early after the diagnosis of AF. Catheter ablation remains the single best therapy for AF; however, it is not free from severe complications and only a small percentage of AF patients in the Western world ultimately receive ablation. Ensuring that AF ablation is safe, effective, and efficient is essential to make it accessible to all patients. With the limitations of traditional thermal ablative energies, pulsed field ablation (PFA) has emerged as a novel non-thermal energy source. PFA targets irreversible electroporation of cardiomyocytes to achieve cell death without damaging adjacent structures. Through its capability to create rapid, selective lesions in myocytes, PFA presents a promising alternative, offering enhanced safety, reduced procedural times, and comparable, if not superior, efficacy to thermal energies. The surge of new evidence makes it challenging to stay updated and understand the possibilities and challenges of PFA. This review aims to summarize the most significant advantages of PFA and how this has translated to the clinical arena, where four different catheters have received CE-market approval for AF ablation. Further research is needed to explore whether adding new ablation targets, previously avoided due to risks associated with thermal energies, to pulmonary vein isolation can improve the efficacy of AF ablation. It also remains to see whether a class effect exists or if different PFA technologies can yield distinct clinical outcomes given that the optimization of PFA parameters has largely been empirical.

## 1. Introduction

Atrial fibrillation (AF) is the most prevalent arrhythmia in humans (it affects 2–4% of Europe’s population and 60 million people globally in 2020) and is associated with increased morbimortality [1,2]. Heart failure and stroke are among the most feared complications associated with AF and they are not completely prevented even when the patient is adequately anticoagulated, and the ventricular rate is well controlled [3,4]. Other comorbidities associated with AF, which are, by no means, less relevant, include cognitive decline, depression, embolic events, an overall poorer quality of life, and reduced life expectancy [5].

## 2. Maintenance of Sinus Rhythm as the Target Therapeutic Goal

Previous studies failed to prove the superiority of rhythm control using antiarrhythmic drugs (AADs) over rate control in hard endpoints, even if a larger proportion of patients remained in NSR [6,7,8,9,10]. However, with the widespread availability of catheter ablation for AF rhythm control, more recent trials have revisited the question of whether rhythm and rate control are comparable. The EAST-AFNET 4 trial demonstrated that rhythm control of early AF (i.e., diagnosed in the year prior to trial enrolment) was associated with a lower risk of adverse cardiovascular outcomes (death from cardiovascular causes, stroke or hospitalization for heart failure or acute coronary syndrome) compared to patients with rate control [4]. These favorable results were driven by a reduction in cardiovascular death and stroke with rhythm control, even when more than half of the patients were in NSR at enrolment and > 90% of patients were receiving oral anticoagulants. The EAST-AFNET 4 trial excluded patients with long-standing atrial fibrillation and included AF catheter ablation as a rhythm control strategy, suggesting that both catheter ablation and reducing “time to rhythm control” are key elements.

Catheter ablation of paroxysmal and persistent AF has proven superior to AADs by improving quality of life and by reducing AF recurrence/burden, cardiac-related hospitalizations, and mortality [11,12,13,14,15,16,17,18,19,20,21]. The latest guidelines for the diagnosis and management of AF sustain a class 1 recommendation for AF catheter ablation of symptomatic AF refractory to AADs, de novo paroxysmal AF or persistent AF with reduced left ventricular ejection fraction [22]. Catheter AF ablation as a first-line therapy for symptomatic persistent AF and for young asymptomatic patients with persistent AF are given a class 2a and class 2b recommendation, respectively [22]. It must be highlighted that the rate of adverse events associated with AF catheter ablation is similar to that reported with the use of AADs [23].

AF is a chronic disease whose natural course is to progress from a paroxysmal to a more persistent presentation; this is estimated to occur in up to 25% of patients after a year of follow-up [23,24,25,26,27]. An oversimplification of the pathophysiological correlate to these clinical stages is the progression from an isolated electrical disorder where firing from pulmonary vein (PV) (or less often, non-PV) foci triggers self-limited AF to a disorder where other non-well-defined mechanisms sustain AF even in the absence of these trigger foci [28]. AF produces several electrical and structural changes that promote its maintenance, explaining the mantra that “AF begets AF” [25,29].

In contrast to AADs, AF catheter ablation can revert AF-induced changes that beget AF and thus prevent disease progression. The ATTEST trial and the EARLY-AF trial demonstrated, respectively, that radiofrequency (RF) and cryoablation for pulmonary vein isolation (PVI) in patients with paroxysmal AF delayed progression to persistent AF [23,30]. Preventing progression is not a trivial objective as persistent AF is associated with an increased risk of thromboembolism, heart failure, hospitalization, and death [26,31,32,33]. Furthermore, AF catheter ablation must be promptly considered given the worse results attained in patients with persistent compared to paroxysmal AF [34,35].

The aforementioned explains that catheter ablation for AF is the most common electrophysiologic procedure in Europe (i.e., on average, AF ablation represents 40% of all ablation procedures) and yet only a small minority of patients with AF are ablated [36]. Despite good-quality data not being easily found for some regions, if we consider only non-permanent AF refractory to anti-arrhythmic drugs, which has a class I indication for catheter ablation, it is estimated that only around 6% of the patients with AF in the Western world receive ablation [36,37]. Therefore, AF ablation procedures need to be expeditious if we aim for the largest number of patients to benefit from this therapy.

## 3. Catheter AF Ablation Needs to Be Safe, Effective and Quick

Until present, the cornerstone of AF ablation is PVI, and it has been performed using thermal energies, essentially RF or cryotherapy, and to a lesser extent, laser. Thermal energies rely on time-dependent conductive heating/cooling, thus causing non-selective ablation of any underlying tissue [36].

While infrequent, RF and cryoablation can have complications, the rate of which is similar among both energy sources, although the type of complication can differ [2]. Pulmonary vein stenosis (PVS), phrenic nerve palsy/paralysis (PNP), atrioesophageal fistula (AEF), stroke, and lesions to other neighboring structures (coronary arteries, vagal nerve) are among the reported complications of ablation with thermal energies. The rates of PVS, PNP, AEF, and stroke or transient ischemic attack are estimated to be, respectively, 0.5%, 2.7%, 0.02 to 0.15% and 0.15 to 0.5% [2,38]. Although these figures can be underscored, the large and increasing number of ablation procedures and the fact that some of these complications can be fatal (83–100% mortality with AEF without repair) cannot be obviated [2].

On average, the total procedure time of RF point-by-point PVI is around 95 min [39,40,41]. Although cryoablation was introduced as a potentially safer and more effective alternative to RF, it proved to be equivalent, except for significantly shorter total procedural time [39,40,42,43].

Beyond being safe and quick, AF ablation needs to be effective. The main mechanism behind arrhythmia recurrence is the reconnection of PVs because of incomplete lesion transmurally and/or contiguity; overall, 73 and 59% of paroxysmal and persistent AF patients remain free from any arrhythmia recurrence after 1 year.

Given the margin for improved safety, efficacy, and procedure duration, the need for a new energy source was evident. Pulsed field ablation (PFA) is a “new” non-thermal energy source that has advantages due to its ability to produce selective lesions to myocytes and its ability to create transmural and contiguous lesions expeditiously with limited collateral damage [44,45].

## 4. Biophysics of Pulsed Field Ablation

**Figure 1:**
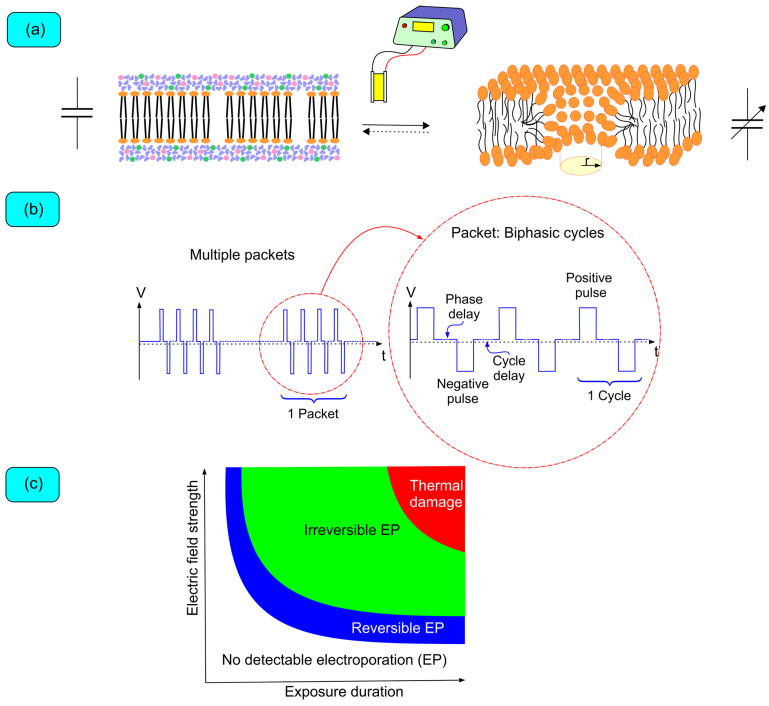
Biophysics of pulsed field ablation. (**a**) The plasma membrane, a bilayer lipid membrane, acts as a capacitor by separating electrical charges (inside: −60 to −70 mV relative to the outside; resting membrane potential). Exposure to an external electric field induces additional charges, creating an induced transmembrane voltage that combines with the resting potential. This voltage facilitates water molecule penetration and phospholipid reorientation, forming aqueous pores. (**b**) Pulsed field generators use direct current power supplies to charge capacitors, which discharge pulses between two electrodes, creating an electric field. Pulses are biphasic, square-shaped, and delivered in bursts. (**c**) Cellular response to the electric field primarily depends on electric field strength and pulse duration.

The first medical application of electroporation began when Neumann et al. published a seminal work on the use of electroporation to temporarily permeabilize mouse cell membranes for gene transfer [46]. Electroporation consists of the formation of transient-lived (nano to milliseconds) aqueous nanopores in the bilayer lipidic cell membranes exposed to a high electric field (Figure 1). These pores are formed when water molecules invade the membrane and lead to reorientation of phospholipids such that their polar heads point toward these water molecules [47].

In resting state, the presence of ionic channels in cell membranes produces a transmembrane voltage gradient, the inside of the cell being negative with respect to the outside.

Cells maintain their internal milieu because the plasma membrane is a selective barrier to the free interchange of molecules between the cytoplasm and external medium. Therefore, when exposed to an electric field, the membrane functions as a capacitor where opposing charges build up on opposite sides and lead to an induced transmembrane voltage (ITV). A cell membrane exposed to an electric field builds up an ITV additional to the resting transmembrane voltage that produces a large number of aqueous pores as its magnitude increases above a critical threshold (between 200 and 500 mV, depending, among other factors, on the cell type and pulse duration) [48]. ITV on single spherical cells can be approximated by the Schwan equation, but it must be noted that this equation is practically of little interest apart from establishing a biophysical understanding [47,49]. The Schwan equation states the following:

ITV = *f*·E·R·cosθ·(1 − e^−t/τ^),

where *f* is a dimensionless factor, *E* is the electric field, *R* is the radius of the cell, *θ* is the angle of the center of the cell with respect to the direction of the electric field, *t* is the time elapsed since the onset of the field, and *τ* is the time constant of membrane charging.

Despite more work being needed to unravel how electroporation produces cell death, it can be conceptualized that the electric field induces changes in cell homeostasis that ultimately produce programmed cell death (apoptosis) or necrosis. During the electric field pulse, membrane permeability increases and transport across the plasma membrane is mainly electrophoretic, mediated by the force exerted on charged particles by the electric field, and electroosmotic, where solutes are dragged by fluid movement across the membrane driven by the electric field. When the cell is no longer exposed to the electric field, and until it reseals, transmembrane transport is essentially dependent on electro-diffusive and endocytosis [47]. The modification in the cellular internal milieu through the alteration in normal transport across the plasma membrane is what triggers cell death.

The electric field is generated by a direct current generator that delivers bursts of rectangular biphasic pulses (there are other pulse configurations, but their discussion is beyond the scope of this review). The pulses can be modified in several ways (Figure 1b): (1) voltage (the greater the field strength, the greater the effect); (2) number of pulses (increased effect with a higher number, although there seems to be a saturation effect); (3) pulse duration (greater effect with longer durations, provided the shortest pulse duration is greater than the time required for ITV build-up); (4) pulse shape; (5) electrode size (greater effect with larger size at the same voltage); (6) inter-electrode distance (greater effect with shorter distance); and (7) stimulation mode (unipolar vs. bipolar; unipolar delivery is associated with deeper lesions but is limited by muscle contraction, which can be prevented with shorter pulses) [50,51,52,53]. Multielectrode catheters for PFA also produce larger lesions than single electrode catheters, based on the 3D shape of the electric field [53]. Optimization of the pulse parameters to better accomplish the ablative application is essentially trial-and-error experimentation, although simulation models are being researched both ex vivo and in vivo [48,49,54,55]. Therefore, quoting Dr. David E. Haines, “once you’ve tried one PFA… you have tried one PFA”.

The effects on myocytes are dependent on many factors; however, the electric field’s strength and the exposure duration remain the most important dosing parameters. The effects of PFA can be as follows: (i) no detectable electroporation, (ii) reversible electroporation, (iii) nonthermal irreversible electroporation, or (iv) irreversible electroporation that is accompanied by thermal effects (Figure 1c). The aim is to achieve nonthermal irreversible electroporation in most of the cell population exposed to the electric field [53]. Depending on the cell size, orientation, and distance from the electrodes, the effect on the population of cells can be heterogeneous. Fortunately, the therapeutic window to achieve irreversible nonthermal electroporation is wide. However, increasing field strength and exposure duration needs to be gauged to avoid excess joule heating of the tissue (heat = resistance · current^2^ · time) [56]. Irreversible nonthermal is always associated with minimal tissue heating (usually <10 °C), the effect of which on the tissue is negligible considering proteins, the most sensitive molecules, start denaturing at approximately 45 °C.

## 5. Advantages of PFA: Tissue Selectivity and Expeditious Energy Delivery

Both RF ablation and cryoablation produce non-selective thermal injuries that do not specifically target the myocardium, putting at risk neighboring anatomic structures [57]. PFA stands out because it produces selective damage to myocardial tissue, which, together with the elimination of pops and charring associated with high temperatures, has been proven to reduce complication rates. In addition to safety improvements, PFA promises less dependency on contact force, shorter procedure times, and the production of more homogeneous lesions.

### 5.1. Tissue Selectivity

**Figure 2:**
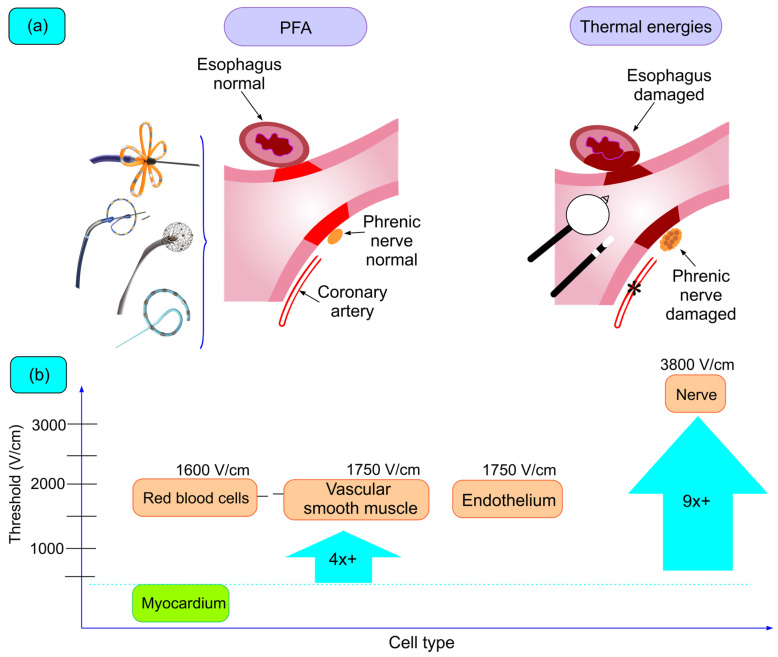
Tissue selectivity of pulsed field ablation (PFA). (**a**) PFA selectively targets atrial myocardium while sparring adjacent structures, contrasting with thermal energy-based methods. (**b**) Electroporation allows for the titration of electric field strength to tissue-specific thresholds, inducing selective cell death. Section (**b**) has been adapted with permission from Elsevier, ref. [38].

The *holy grail* of PFA is myocardial tissue selectivity, which contrasts with thermal ablation technologies (Figure 2). The tissue selectivity of PFA relies on the significantly lower threshold to PFA of cardiomyocytes compared to other tissue types and is related to the high electrical conductivity and larger size of myocytes. There is a large body of preclinical evidence demonstrating the safety of PFA; even when deliberately applying PFA on relevant structures like the esophagus or the phrenic nerve, no lesions were detected (Figure 2a) [58,59,60,61].

Despite its questionable extrapolation to human mature cardiomyocytes, a frequently cited paper to evaluate the differential sensibility of PFA used rat cardiomycytes. In this work, electroporation demonstrated that after application of electrical fields of >375 V/cm cell proliferation decreased significantly compared to lower field strengths [62]. This viability threshold was significantly lower than that of other tissues studied with the same protocol, indicating that a careful titration of energy could produce damage to cardiomyocytes while sparring other tissues from being injured (i.e., electric field strength within the range of 400 V/cm to 1200 V/cm is considered adequate to induce electroporation while minimizing damage to neighboring structures) (Figure 2b [53]. There are other works evaluating electroporation thresholds for cardiac tissue, but the different study methodologies (particularly cells from different species) make comparisons difficult. There is still limited knowledge of the PFA threshold on other tissues of interest during cardiac arrhythmia ablation. The major complications associated with thermal ablative energies include PVS, lesions to neighboring structures, and stroke. Evidence suggesting the safety profile of PFA is presented later in this review with the results of clinical trials. In this section, we will succinctly mention some evidence from work specifically conceived to study the comparative safety of RF and PFA.

#### 5.1.1. Esophageal Lesions

A work by Cochet et al. evaluated esophageal injuries after PFA of paroxysmal AF with cardiac magnetic resonance (MRI) [62]. Compared with preprocedural MRI, an acute study performed < 3 h post procedure showed no esophageal lesions in patients ablated with PFA. However, patients undergoing ablation with thermal methods presented lesions in up to 43% of cases despite the fact the rate of direct contact between the esophagus and the ablation sites was similar between cohorts. Three months post procedure, no lesions were detected in the esophagus in any of the patients, irrespective of the ablation energy used. This same work evaluated acute lesions on the descending aorta and found no difference in the rate of lesions between RF and PFA (43 vs. 33%; *p* = 0.52), suggesting the PFA threshold of aortic tissue might not be as low as expected. Like with esophageal lesions, no patient showed evidence of aortic lesion at 3 months. This work suggests the safety of applying near the left atrium posterior wall, paving the way to isolating the posterior wall.

#### 5.1.2. Nervous Lesions

Stojadinovic et al. evaluated the mechanisms of PFA and analyzed the changes evoked in the autonomic nervous system after PFA and RF ablation using extracardiac vagal stimulation [62]. The vagal response of both the sinoatrial node and the atrioventricular node was significantly attenuated with RF ablation at the end of PVI when compared with PFA. Sinus rhythm also experiences a greater acceleration after RF ablation. There is evidence of intraprocedural nerve stunning that recovers by procedural completion in all but a third of patients undergoing PFA, which have a reduced vagal response. Although it has been speculated that parasympathetic efferent denervation may reduce arrhythmic recurrences, it remains to be determined whether this might impair PFA results or is just an epiphenomenon of younger patients, less prone to AF recurrences and higher parasympathetic modulation [63,64,65]. This work suggests that PFA relatively spares ganglionated plexi and larger nerves, like the phrenic nerve, even if they can be captured during PFA applications [66,67].

#### 5.1.3. Vascular Lesions (Pulmonary Veins and Coronary Arteries)

Preclinical evidence with animal models demonstrated that even when deliberately trying to injure PVs with PFA, this was not possible [59]. The ADVENT trial, which is presented later, was the first randomized controlled trial comparing PFA and thermal ablation [40]. There were no cases of PVS; however, the change in the PV cross-sectional area was significantly lower with PFA (−0.9% vs. −12%; posterior Bayesian probability > 0.999) [68]. Another work including data from different clinical trials using PFA or RF ablation in which CT scans were available concluded that ostial diameters were significantly reduced with RF ablation [68]. Mild, moderate, and severe PV narrowing was observed in 9, 1.8, and 1.2% of patients in the RF cohort; no PV narrowing was documented with PFA.

Preclinical data showed cases of reversible coronary spasms, the mechanism of which is unclear, and this has been documented in the postmarked clinical arena using different PFA systems (although there seems to be some publication bias in this respect) [69]. Available evidence comes from case reports or observational studies, where the application of PFA near the coronary arteries (circumflex artery with mitral isthmus or right coronary artery with cavotricuspid isthmus) leads to reversible coronary spams [70]. A prospective trial including 26 patients receiving the first PFA or RF ablation of the mitral isthmus evaluated coronary spasm before, during, and after ablation [71]. In this study, 7 of 17 patients receiving PFA experienced coronary spasms (only two of which required treatment with nitroglycerin); this only occurred with an anterior mitral line. No coronary spasm has been reported in patients receiving RF. Although subclinical and rarely leading to complications, coronary spasms can be prevented by pretreating with nitroglycerin (repeated IV boluses or IV continuous infusion) [72]. Limited evidence suggests PFA-induced spasms (when performing applications in the endo and epicardium) do not produce new coronary lesions in the short term [73,74].

#### 5.1.4. Cerebral Lesions

Some preclinical work demonstrated gas bubble formation after PFA but failed to document MRI or histologic cerebral lesions [75]. However, the reported rate of silent cerebral injury across several clinical studies using PFA falls within the reported incidence (between 4 and 45%) of these lesions when using thermal energies [73]. It is suggested that AF catheter ablation may reduce the risk of neurocognitive deterioration; however, it is still a matter of debate how is this balanced with the appearance of subclinical lesions after the ablation procedure, albeit many resolve with time [76]. Further research is needed in this respect with PFA; however, in terms of clinically relevant cerebral events, this technology has proved safe (0.11 and 0.4% incidence of transient ischemic event and stroke, respectively) [77].

### 5.2. Procedural Technical Advantages

**Figure 3:**
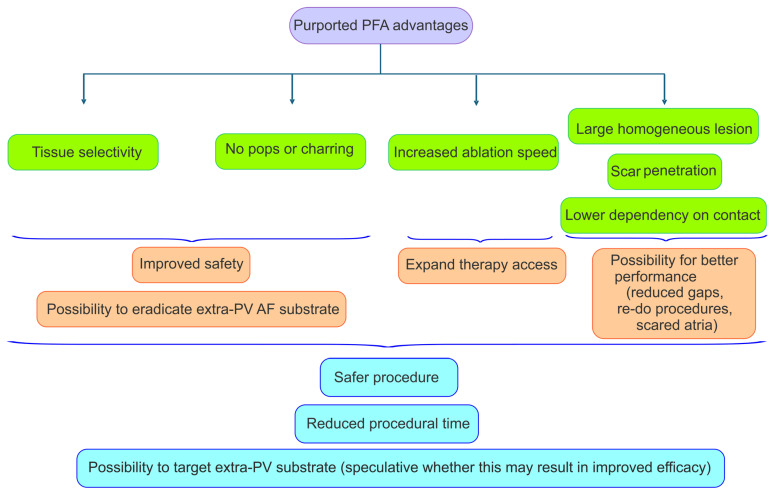
Purported advantages of pulsed field ablation (PFA). PFA’s tissue selectivity has been shown to minimize damage to adjacent structures, thereby reducing energy-specific risks associated with atrial fibrillation (AF) catheter ablation. From a technical perspective, PFA does not significantly increase tissue temperature, eliminating the risk of charring and pops, a characteristic that may enhance procedural safety. Additionally, PFA creates uniform lesions, regardless of the presence of scar tissue, and demonstrates less dependence on contact force. Clinical trials with a dual-arm design comparing PFA with thermal ablation have demonstrated that PFA is safer and reduces overall procedural times. It remains speculative whether future refinements in PFA delivery will improve the efficacy of AF ablation.

Evaluating procedural times within the context of clinical trials is challenging as they often incorporate mapping before and after ablation or necessitate a waiting period to confirm PVI (Figure 3). However, the most extensive survey to date on the use of the single-shot Farawave™ catheter (Boston Scientific, Marlborough, MA, USA) reports a mean procedural time of 65 min, which includes in some cases pre- and/or post-ablation electroanatomical mapping [77]. In the insPIRE trial testing the Varipulse™ catheter (Biosense Webster, Irvine, CA, USA), where there was no need for a waiting period to check for PVI and where up to 77% of patients were mapped, the total procedural time was 70 min [78]. These times, when compared with other registry data utilizing cryoablation, are notably better, especially considering the novelty of the PFA technology. Reported real-world total procedural times for cryoablation range from 84 to 113 min [42,43,78]. Non-randomized studies comparing PFA and cryo-balloon may show differing results in terms of efficacy (PFA being equal or superior); however, they all report shorter procedural times with PFA [79,80].

During RF ablation, high blood flow through the area of interest results in significant heat dissipation, typically managed by increasing power output. This escalation, however, raises the risk of mechanical complications, notably steam pops and cardiac perforation. Additionally, the irrigation of RF catheters can cool the tissue-catheter interface, potentially leaving a rim of viable endocardial tissue (gaps). PFA circumvents these issues.

While optimal electrode–tissue contact remains crucial, particularly in tissues with irregular surfaces where contact may be suboptimal, PFA has demonstrated the ability to produce more uniform and homogeneous lesions compared to those generated using RF energy [81]. It is important to note that the strength of the electric field diminishes inversely with the square of the distance from the electrodes. Therefore, while tissue contact is mandatory for cryoballoons to achieve PVI, PFA can produce lesions even when there is no contact (although with a reduced lesion size) [79]. This may explain why non-randomized studies comparing these technologies demonstrate higher short-term PVI with PFA [79].

While PFA improves when the catheter is in contact with the target tissue, it does not seem to require the application of contact force as much as RF [81,82]. Preclinical evidence in ventricular myocardium indicates that lesion size, particularly lesion depth, increases with enhanced contact, though to a lesser extent than with RF ablation [82]. Achieving good tissue contact is not only a matter of efficacy in producing lesions but also a matter of safety. Intravascular hemolysis, frequently associated with PFA, but very rarely producing acute kidney injury, is reduced with enhanced catheter contact [83]. Experimental studies have shown that hemolysis increases linearly with repeated pulsed field applications, with a more pronounced effect when catheter–tissue contact is insufficient, reflecting the greater exposure of blood to the electrical field [84]. Other factors including dehydration, hypotension, pre-existing renal disease and the performance of a CT scan using iodinated contrast medium the days before the PFA can predispose patients to hemolysis-induced acute renal failure [85].

Another notable advantage of PFA over RF ablation is its ability to generate transmural lesions in scarred myocardial areas. Current evidence predominantly arises from preclinical studies conducted on ventricular myocardium in swine models [86,87]. These studies demonstrate that PFA induces epicardial ablative lesions in regions with ischemic myocardial scars. In contrast, the RF effect is confined to the endocardium due to its limited capability to achieve high intramyocardial temperatures. While this hypothesis remains speculative, it may hold significant implications for AF ablation in patients with severely diseased atria or in re-do procedures.

## 6. Clinical Experience

**Figure 4:**
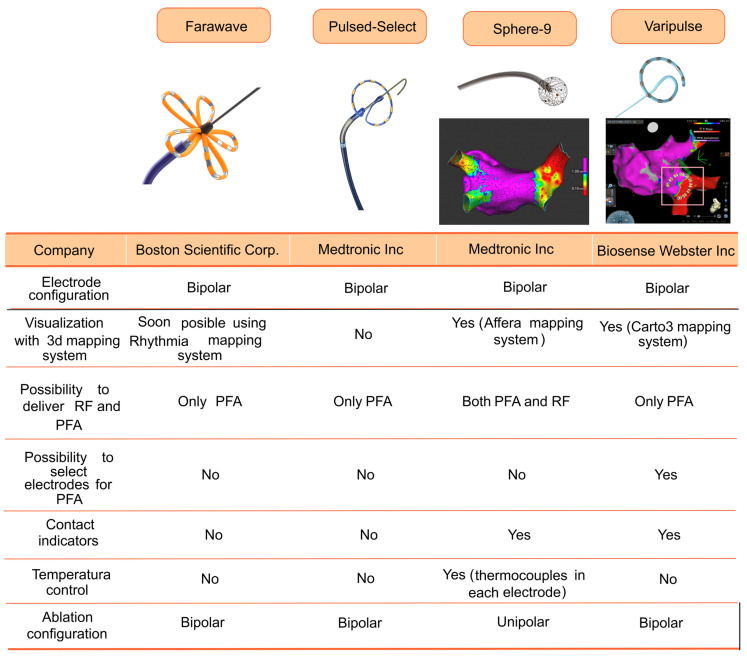
Catheters capable of pulsed field ablation that have received CE market approval. Comparison of different characteristics associated with each catheter.

Preclinical experience in swine and canines demonstrated PFA could produce electrical isolation of the PVs and the atrial appendages, sparring neighboring structures [88]. These experiments also demonstrated that PFA waveform modification could alter the long-term efficacy, leading the way to clinical experience where every PFA catheter had its own “secret” waveform parameters (Figure 4).

The first clinical experience with PFA was documented in a prospective, single-arm study published in 2018 (Table 1), which involved 15 patients with paroxysmal AF candidates for endocardial catheter ablation [89]. This study used the 12-F over-the-wire Farawave™ catheter (Boston Scientific, Marlborough, MA, USA), which consists of five splines, each equipped with four electrodes (one of them able to sense/pace), and that can be basket- or flower-shaped. This work demonstrated acute efficacy (100% first-pass PVI and no evidence of acute reconnections) with no acute catheter-related complications. No follow-up was performed.

With the purpose of expanding the use of PFA beyond PVI in persistent AF, a later trial using the Farawave™ catheter included 25 patients with symptomatic persistent AF refractory to anti-arrhythmic drugs, aiming for PVI and posterior wall ablation [90]. Targeting the electrical isolation of the left atrial posterior wall has shown variable results when using thermal energies, possibly hampered by the fear of producing durable lesions at the cost of an increased risk of AEF. Posterior wall isolation was revisited with this trial, where approximately 3 months post procedure, 96% of PVs were successfully isolated, and complete posterior wall isolation was achieved in all patients, despite lesion size shrinkage in three cases. No safety concerns were reported with the PFA system. This study suggests that expanding ablation targets in persistent AF using PFA is possible without compromising patient safety. It remains to be proven if this can lead to improved efficacy.

In 2021, data were published from a cohort of 121 patients participating in three closely related prospective single-arm trials assessing the effect of PFA with the Farawave™ catheter in patients with paroxysmal AF [91].

**Table 1:** Clinical trials of PFA catheters with CE market approval. Abbreviations: AEF = atrioesophageal fistula; CT = computed tomography; F/U PVI = follow-up pulmonary vein isolation; GA = general anesthesia; MRI = magnetic resonance imaging; PNP = phrenic nerve palsy; pts = patients; PVS = pulmonary vein stenosis.

Study	Catheter	N	Paroxysmal (%)	F/U PVI%	Clinical Efficacy	Complications	Anesthesia
2018, Reddy [89]	Farawave	15	100%	Not evaluated	Not evaluated	No acute complications. No long-term studies.	GA
2020, Reddy [92]	Sphere-9	76	72%	Not evaluated		One groin hematoma. No AEF (60 pts with esophagoscopy), PVS (44 pts with CT) or PNP. 5/51 pts with silent cerebral lesions/events.	GA (100%)
2020, Reddy [90]	Farawave	25	0%	22 pts remapped at 3 mon. 86% of pts. *Posterior wall isolated in 100%.	Not evaluated	1 effusion with RF-based remapping. No AEF (21 pts with endoscopic study), PVS (14 pts with CT) or PNP.	80% conscious sedation; 20% GA
2021, Reddy [91]	Farawave	121	100%	110 pts remapped 3 mon. 65% of pts. (84% with optimized waveform)	(excluding repeat procedure): 79%. Optimized waveform cohort (44 pts): 85%	2 cardiac perforations and 1 vascular complication. No AEF (38 pts with endoscopic study), stroke (18 pts with MRI), PVS (74 pts with CT or MRI) or PNP. 2/18 pts evaluated with MRI had acute lesions.	GA for monophasic (15/15). Sedation for almost all biphasic (105/106)
2021, Verma [44,45]	Pulsed-select	38	92%	Not evaluated		No acute complications. No long-term studies.	GA (76%); conscious sedation
2023, Verma [93]	Pulsed-Select	300	50%	Not evaluated	70% paroxysmal, 62% persistent	1 effusion and 1 cerebrovascular accident. No AEF, PVS (63 pts with MRI) or PNP. 4/45 pts evaluated with MRI had silent cerebral lesions.	GA or deep sedation in 95% of paroxysmal cohort and 92% persistent cohort
2023, Reddy [94]	Sphere-9	178	39%	122 pts remapped at 3 mon. 58% of patients (90% of pts. with optimized waveform)	78% (for combined paroxysmal and persistent)	1 inflammatory pericardial effusion medically treated. No AEF (124 pts with esophagoscopy), PVS (77 pts with CT) or PNP. 1 groin hematoma. 13/89 pts with silent cerebral lesions/events.	GA (100%)
2024, Duytschaever [78]	Varipulse	226	100%	Not evaluated	76.9% and 78.9% for wave I and wave II	No AEF, PVS or PN injury. 8/39 pts with silent cerebral lesions/events; all resolved at 3 months post procedure.	Conscious sedation (27%) or GA (73%)

The results of the overall cohort must be interpreted with caution due to the use of different PFA waveforms during the trials (15 patients were treated with a monophasic PFA waveform, and 106 patients with a biphasic waveform, of which 49 received an optimized biphasic PFA).

After a one-year follow-up, freedom from any atrial arrhythmia (excluding the blanking period) in the entire trial cohort after a single procedure was 79%. Among patients treated with the optimized biphasic waveform, this percentage increased to 85%. A substantial portion of the patients (91%) underwent a second procedure to evaluate PVI 3 months post-procedure, with 65% of patients from the entire cohort exhibiting at least one reconnected PV (84% among those treated with the optimized biphasic waveform). Analyses circumscribed to patients treated with the optimized waveform were post hoc and, thus, should be interpreted with caution.

The primary safety endpoint occurred in 2.5% of patients (two cardiac perforations and one vascular hematoma), with no damage to neighboring structures. Out of the 18 patients who underwent a brain MRI, no lesions were detected in 16 (89%).

The results of this compound cohort indicate that PFA compares favorably with thermal energy ablations in terms of lesion durability and clinical efficacy without causing damage to adjacent structures.

After the Farawave™ catheter achieved its CE market approval in 2021, the Pulsed-Select™ catheter (Medtronic Inc., Minneapolis, MN, USA) was the second single-shot PFA catheter to receive CE market approval in 2023 based on the prospective, multicenter, single-armed PULSED AF pilot and pivotal trials [44,93]. The PULSED AF pivotal study recruited patients with symptomatic paroxysmal (n = 150) or persistent (n = 150) AF refractory to anti-arrhythmic drugs [93]. The Pulsed-Select™ catheter, reminiscent of the PVAC-Gold™ catheter (Medtronic Inc, Minneapolis, MA, USA), is a nine-electrode circular array catheter capable of sensing, pacing, and ablating applying a biphasic bipolar waveform. Acute PVI was achieved in 100% of patients and the primary safety endpoint occurred in one patient in each cohort (pericardial effusion and cerebrovascular accident). A total of 4 out of 15 patients had silent cerebral lesions. The 1-year treatment success, defined as freedom from a composite of multiple failure modes, was 66.2% and 55% in the paroxysmal and persistent AF cohort. For comparison with other trials, the 1-year freedom from any atrial arrhythmia in these cohorts was 70 and 62%, respectively, and resulted in an increase in patient quality of life. In contrast to the Farawave™ trials, the PULSED AF had fewer patients ablated during the blanking period (2% vs. 32%—it must be clarified that the Farawave™ trials prespecified a remapping procedure after the blanking period) [91]. The Pulsed-Select™ catheter is comparable in terms of efficacy with other PFA systems and with other trials using thermal ablation energies.

**Figure 5:**
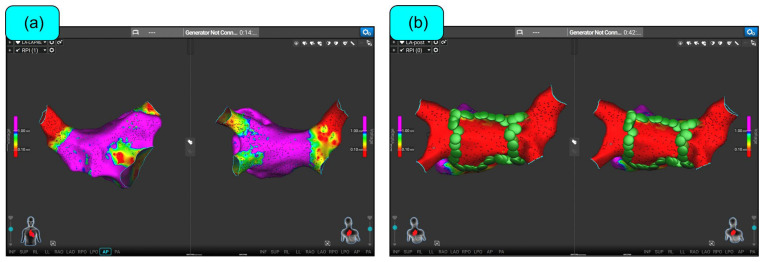
Affera™ (Medtronic Inc., MN, USA) 3D mapping system. (**a**) Pre-ablation voltage map of the left atrium. (**b**) Post-ablation voltage map of the left atrium. Green circles indicate the focal footprint of the Sphere-9™ catheter used for pulsed field ablation. The patient underwent wide-circumferential antral ablation and posterior box isolation with both superior and inferior lines. Source: routine ablation performed at our center outside an investigational context when the Sphere-9 catheter had already received CE market approval.

All catheters mentioned are intended for single-shot ablation of AF, limiting the delivery of extra-PV lesions. Sphere-9™ (Medtronic Inc., Minneapolis, MN, USA) is an 8F 9 mm compressible lattice-tip catheter capable of delivering large-focal RF or PFA lesions supported by the Affera™ (Medtronic Inc., Minneapolis, MN, USA) mapping system (Figure 5). There are nine mini-electrodes with integrated thermocouples on the lattice surface and a central indifferent electrode. Bipolar electrograms were configured between each mini-electrode and the central electrode. The first-in-human single-arm multicenter trial evaluated the use of this catheter for PVI in both persistent and paroxysmal AF using a strategy of either PFA posteriorly and RF anteriorly (PFA/RF cohort) or PFA throughout as per operator preference [92]. The lattice can deliver a biphasic unipolar pulsed field waveform or temperature-controlled RF with just a change in the generator configuration, allowing for the synergistic utilization of both energy types. All PVs were acutely isolated, although it needs to be mentioned that 19% of PV pairs were reconnected after a 20 min waiting period and required additional ablation. There were no major complications, specifically no stroke, PNP, PVS stenosis, or AEF. It should be mentioned that two patients had minor erythema detected in esophagogastroscopy and they were all from the PFA/RF cohort, where RF was inadvertently applied to the posterior wall. There were 3 out of 51 patients (5.9%) with silent cerebral lesions. Among patients with persistent AF, the operator could deliver additional linear ablations (mitral isthmus, cavotricuspid isthmus, etc.), achieving conduction block in 98.9% of lines using exclusively the Sphere-9™. Importantly, posterior mitral isthmus line block was achieved in 100% of cases (in 13 out of 14 cases only the trial catheter was used; in one case, the use of a standard irrigated radiofrequency catheter was required).

A later study using the Sphere-9™ catheter assessed lesion durability with remapping procedures and evaluated clinical efficacy after 1 year of follow-up [94]. This prospective, single-arm trial included patients with symptomatic paroxysmal and persistent AF. Acute PVI was achieved in all cases with 95% first-pass isolation. At 3 months post procedure, 122 of 178 patients were remapped. The authors observed that 75% of PVs were isolated with 58% of patients having all PVs isolated. The 1-year freedom from any atrial arrhythmia was 78.3% and 77.9% for the paroxysmal and persistent AF cohorts, respectively. These data are comparable to the clinical efficacy achieved with the Farawave™ catheter. However, they need to be interpreted considering that the trial allowed for the delivery of additional optional linear lesions (78 patients with mitral isthmus lines, 130 with a roof line and 38 with a posterior inferior line) and the fact that the PFA waveform in the persistent cohort was mostly an optimized version of that used in the paroxysmal cohort. The PFA waveform was modified 3 times during the study, with the latest optimization leading to a significantly lower number of acute PV reconnexions, greater first-pass isolation, more durable lesions (the per-vein durability rate increased from 51 to 97%) and greater 1-year freedom from atrial arrhythmias when compared with the first waveform parameters. These findings were also observed with the Farawave™ catheter when comparing the optimized with the initial waveform [91]. The only adverse effect was an inflammatory pericardial effusion that was medically managed.

The ADVENT trial was the first multicenter randomized trial demonstrating non-inferiority of PFA using the Farawave™ catheter in comparison to conventional thermal ablative technologies in patients with paroxysmal AF [40]. A total of 305 patients were allocated to the PFA group and 302 underwent thermal ablation (approximately 50% with cryoballoon). At 1 year follow-up, the primary endpoint of freedom from a composite of initial procedural failure, recurrence of any atrial arrhythmia or need to implement any rhythm control strategy after the blanking period were non-inferior in the PFA group (probability of treatment success in the PFA and RF groups was of 73% and 71%, respectively; lower boundary of the 95% Bayesian credible interval was of −5.2% with an accepted margin for non-inferiority of 8%; posterior probability of non-inferiority > 0.999). The primary safety endpoint was also non-inferior in the PFA group (2.1% vs. 1.5%; the lower boundary of the 95% Bayesian credible interval was −1.5% with an accepted margin for non-inferiority of 15%; posterior probability for non-inferiority > 0.999). There were no AEF or PVS in either group, although there were two patients with persistent PNP caused by cryoballoon ablation. Procedural time was shorter in the PFA group (106 vs. 123 min; 95% Bayesian credible interval: −23.1 to −11.5), although at the expense of increased fluoroscopy time (21 vs. 14 min; 95% Bayesian credible interval: −5.2 to 9.1 min).

The results from the ADVENT trial are in accordance with those from non-randomized studies comparing PFA using the Farawave™ catheter with the Artic Front Advance 28 mm cryoballoon (Medtronic Inc., MN, USA) [79,80]. These studies also demonstrated shorter procedural times, no evidence of PN injury, and equal or improved 1-year clinical efficacy in the PFA group.

The aforementioned results are also in line with real-world outcomes, as demonstrated in the EU-PORIA and MANIFEST registries studying a total of 1233 and 1568 patients, respectively, considered for AF ablation with the Farawave™ catheter [66,67]. These registries demonstrate an acute PVI of >99% with a mean total procedural time of 58–61 min. The 1-year AF-free survival was 80–82% and 66–72% for the paroxysmal and persistent cohorts. The rate of major complications was 1.7–1.9% mainly driven by pericardial tamponade. Minor complications occurred in 1.9–4.0% of patients, mostly related to vascular access site complications. Only one PNP did not recover by the end of the procedure and had an injury persisting beyond 1 year. Data from the MANIFEST-17K registry, including >17,000 consecutive patients undergoing AF ablation (57.8% paroxysmal) with the Farawave™ catheter in 106 different centers with >400 different operators, are in line with previous results [85]. There were no cases of AEF, PVS or persistent PNP with an overall rate of major non-PFA-specific complications of 0.98% (mainly driven by cardiac tamponade and access site complications; 0.36% and 0.30%, respectively). It should be mentioned that vasospasm and acute renal failure due to hemolysis were reported in 0.14 and 0.03% of patients, respectively. Patients experiencing renal failure received a mean of 143 PFA applications. It is interesting to mention that deep sedation was employed in 56% of cases in favor of general anesthesia.

A study with 181 real-world consecutive patients from the SWISS-AF-PVI registry undergoing ablation for paroxysmal or persistent AF using PFA (Farawave™ catheter) or the PolarX™ cryoballoon (Boston Scientific, MA, USA) did not find differences in terms of AF recurrence after 1 year (76 vs. 70%, *p* = 0.99) [88]. There were no statistically significant differences in procedural complications between cohorts. There were no cases of AEF, stroke, or PVS; however, there were three cases of PNP in the cryoballoon cohort and none in the PFA cohort. Interestingly, the total procedure duration was not significantly different between groups (approximately 56 min), but the PFA group used a mapping system (CARTO3; Biosense Webster, Irvine, CA, USA) to confirm PVI isolation. Omitting the use of 3D mapping would possibly have made PFA procedural time shorter, in concordance with the results from a single-center retrospective study comparing the Arctic Front Advance cryoballoon (Medtronic Inc., Minneapolis, MN, USA) with PFA [88].

The SPHERE Per-AF was a multicenter, randomized, non-inferiority trial recently published comparing the Sphere-9 with conventional RF ablation (Thermocool Smarttouch and CARTO3; Biosense Webster, Irvine, CA, USA) for the treatment of persistent AF [95]. A total of 432 patients were randomized to the PFA (219 patients) or to the RF group. Although discouraged, most patients in both arms received additional linear lesions (96 and 85% of patients in the PFA and RF groups, respectively). At 1 year follow-up, the primary endpoint of freedom from a composite of initial procedural failure, recurrence of any atrial arrhythmia, repeat ablation or need to implement any rhythm control strategy after the blanking period were non-inferior in the PFA group (probability of treatment success in the PFA and RF groups was of 74% and 66%, respectively; lower boundary of the 95% Bayesian credible interval was of −0.9% with an accepted margin for non-inferiority of 8%; posterior probability of non-inferiority > 0.999). A post hoc analysis to evaluate the effect of mitral and roof lines revealed no apparent effect on the clinical efficacy. The primary safety endpoint was also non-inferior in the PFA group (1.4% vs. 1.0%; the lower boundary of the 95% Bayesian credible interval was −2.8% with an accepted margin for non-inferiority of 15%; posterior probability of non-inferiority > 0.999). There were no reports of AEF, PVS or permanent PNP. The total procedural time was significantly shorter in the PFA group (101 vs. 126 min, 95% Bayesian credible interval: −33.0 to −17.3 min) not only because of the speed of energy delivery but also because the Sphere-9 is used for mapping and ablation, obviating the need for two separate catheters (and thus transeptal punctures or catheter exchanges).

**Figure 6:**
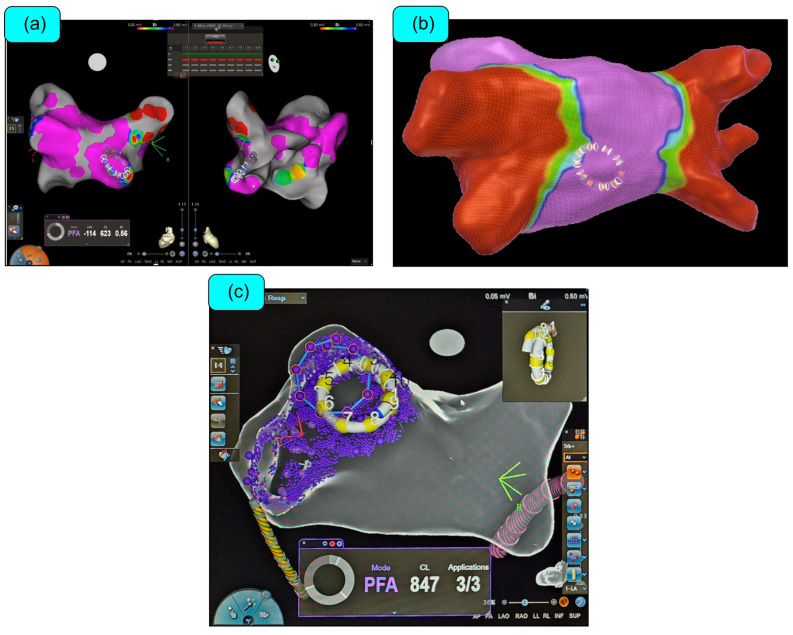
Varipulse™ integrated into the CARTO3™ (Biosense Webster, Irvine, CA, USA) mapping system. (**a**,**b**) The Varipulse™ catheter is moved to create electroanatomical map of the left atrium. The catheter delivers pulsed field ablations (PFA) at precise locations through accurate visualization. The tissue proximity indicator (electrodes bordered in thicker white lines in (**a**)) is used to evaluate the contact with the endocardium. Pulsed field tag coloring provides information of the ablation; inter-tag connectors reveal lesion contiguity between electrodes (**c**). Left atrial final voltage map acquired after PFA (**b**). Source: image provided by Biosense Webster.

The last PFA catheter to receive CE-mark approval has been the Varipulse™ variable-loop catheter (Biosense Webster, Irvine, CA, USA). Like the Sphere-9™ catheter (and soon the Farawave™ catheter), which can be visualized with a mapping system, this catheter can be visualized with the 3D Carto3 mapping system (Biosense Webster, Irvine, CA, USA) (Figure 6).

The parameters of the PFA delivered by the 10-electrode Varipulse™ catheter have been, like with other PFA systems, tested and optimized via bench and preclinical testing and kept confidential. All electrodes can be used for visualization, sensing, pacing, and ablation; in the latter case, like with the PVAC-Gold™ catheter (Medtronic Inc., Minneapolis, MN, USA), in the event electrodes are in close contact, they can be turned off. The Varipulse™ catheter was tested in the inspIRE prospective, multicenter, single-arm clinical trial enrolling patients with symptomatic paroxysmal AF [78]. There were no reports of adverse events (0% in contrast with the PULSED AF trial, Sphere-9 feasibility trial, ADVENT trial and EU-PORIA registry were 0.7%, 0.6%, 2.1% and 3.6%, respectively) [40,67,93,94]. Thirty-nine patients were specifically studied with pre- and post-ablation cerebral MRI, detecting subclinical lesions in 8 patients, all of which resolved after 3 months.

The Varipulse™ catheter managed acute PVI in all cases, without acute reconnection in 96–97.1% of patients. The 1-year freedom from atrial arrhythmia episodes after a 3-month blanking period was 76.9% and 78.9% with the first and optimized PF wave parameters, respectively. Stringent monitoring strategies to detect arrhythmia recurrences, like in the aforementioned trials, were used. However, in contrast with previous trials with Farawave™ and Sphere-9™ trials, where there was a per-protocol mandated remapping at 3 months (and thus a second chance for ablation if there was no durable PVI), the inspIRE trial did not include an invasive PVI evaluation at follow-up [91,94]. The advantage of incorporating catheter visualization in a 3D mapping system strays in the possibility of aiming for a zero-fluoroscopy procedure and allows researchers to deliver PFA to completely encircle veins. Compared with the Pulsed-Select™ and the Farawave™, where the total fluoroscopy time was above 20 min (26 min in the PULSE AF trial and 21 min in the ADVENT trial), the fully integrated platforms for 3D electroanatomical mapping of the Sphere-9™ and the Varipulse™ catheters have proven to substantially reduce fluoroscopy time (4 min in the Sphere-9™ feasibility trial and 3.5 min in the inspIRE trial [40,78,93,94]. The prospective, single-arm, NAVIGATE-PF study of the Faraview™ software module that will enable the visualization and tracking of the Farawave catheter has been initiated. It is expected that soon (possibly by the end of 2024) the Farawave™ catheter will also be integrated into a 3D mapping system.

As of the time of this review, there are four catheters marketed in Europe for performing AF ablation using PFA. None of these catheters have been compared to each other; however, despite differences in their clinical studies, they have shown to provide speed and safety to the ablation procedure with similar efficacy to that achieved with cryoballoons and radiofrequency catheters. It is likely that PFA will replace thermal ablation energies, and it is expected that there will be a surge in new catheters marketed in the near future.

## 7. Future Directions

The development of catheters for PFA of AF has evolved rapidly. This is understandable given that AF is the most common arrhythmia and consequently the most frequent substrate ablated in electrophysiology laboratories. Nevertheless, the precise mechanisms through which electroporation induces “clean” specific myocardial cell death remain to be fully understood. Future research should focus on elucidating and clarifying these mechanisms.

Additionally, investigating the potential synergy between thermal energies and PFA is crucial. Specifically, studies should examine whether combining these modalities can enhance ablation efficacy. Another important area of study involves the treatment of persistent AF, where mechanistic uncertainties persist. It is essential to determine whether the capability of PFA to safely ablate regions beyond the PV will improve AF ablation outcomes. Likewise, it will be key to assess the effect of PFA on scar tissue, which is of particular interest in re-do procedures or in the presence of scars.

Lastly, comparative studies of different PFA catheters are needed to ascertain whether a class effect exists or if different PFA technologies may have different clinical results. Addressing these research gaps will be vital for optimizing the application of PFA in AF management and improving patient outcomes.
